# Ultrasound‐Actuated Gene Editing in Human Kidney Organoids

**DOI:** 10.1002/advs.202520402

**Published:** 2026-06-15

**Authors:** Michael A. Miller, Nicole Vo, Utkarsh, Ivan Sokirniy, Justin Pritchard, Benjamin S. Freedman, Scott H. Medina

**Affiliations:** ^1^ Department of Biomedical Engineering Pennsylvania State University University Park Pennsylvania USA; ^2^ Division of Nephrology University of Washington School of Medicine Seattle Washington USA; ^3^ Kidney Research Institute University of Washington School of Medicine Seattle Washington USA; ^4^ Institute for Stem Cell and Regenerative Medicine University of Washington School of Medicine Seattle Washington USA; ^5^ Department of Medicine University of Washington School of Medicine Seattle Washington USA; ^6^ Molecular, Cellular, and Integrative Biosciences Program Pennsylvania State University, University Park Pennsylvania USA; ^7^ Huck Institutes of the Life Sciences Pennsylvania State University University Park Pennsylvania USA; ^8^ Department of Laboratory Medicine & Pathology University of Washington School of Medicine Seattle Washington USA; ^9^ Department of Bioengineering University of Washington Seattle Washington USA; ^10^ Plurexa LLC Seattle Washington USA; ^11^ Center for Biodevices Pennsylvania State University University Park Pennsylvania USA

**Keywords:** bioacoustics, CRISPR delivery, non‐viral genome editing, organoids, supramolecular assembly

## Abstract

Efficient delivery of gene editing ribonucleoproteins (RNPs) into the interior of solid tissues remains a key hurdle to the clinical translation of non‐viral CRISPR‐Cas9 technologies. Here, we report acoustically‐actuated peptide nanoemulsions (NPeps) that can be spatiotemporally guided and activated by ultrasound to ballistically deliver RNPs into cells within the bulk of dense 3D cellular structures. Using human kidney organoids as a model, we demonstrate NPep vectors improve the spatial profile of gene editing in the organoid mass relative to commercial lipofection reagents, without disruption of tissue structure or qualitative viability features. This technologic paradigm is poised to advance imaging‐guided, deep tissue RNP delivery modalities to expand the clinical diagnostic and therapeutic potential of CRISPR‐Cas9 editing strategies.

## Graphical abstract

Peptide‐stabilized nanoemulsions enable spatially controlled, ultrasound‐triggered delivery of gene‐editing proteins deep within human kidney organoid tissues. Focused acoustic pulses drive droplet vaporization, propelling cargo into target renal cells while preserving native tissue microarchitecture. This non‐viral platform improves both the depth and consistency of gene editing, offering a translatable strategy for precision genome modification in complex 3D organs.

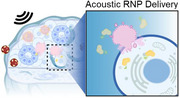

## 1 Introduction

Organoids derived from human pluripotent stem cells represent an integral component in the modeling spectrum of human disease and tissue development. These 3D culture systems can recapitulate the unique architecture and cellular organization of human tissues, thus establishing them as an important bridge between 2D in vitro systems and in vivo animal testing [1, 2, 3, 4]. Importantly, genetic mutations can be installed at the start of stem cell differentiation, and/or during organoid development, to mimic the various stages of disease pathology and tissue reconstruction [2, 5, 6, 7, 8]. Yet, appropriately modeling these genetic disorders, as well as performing genomic screening and establishing reporter systems, requires methods to affect specific genetic mutations and repairs within organoids, and disease‐specific cell types, while keeping the microstructure intact.

While viral gene delivery methods demonstrate high transduction efficiencies, their deployment is restricted by biosafety and mutagenesis concerns, limitations on DNA vector size, and poor virion diffusion through the dense and compact organoid structure [9]. Although non‐viral approaches, including electroporation and lipofection, address some of the safety concerns inherent to viral vectors, they too suffer from poor tissue diffusion and, as a result, low transfection efficiencies (<5%–20%) [9]. To circumvent this, organoids are often dissociated into single cells or cell clusters, non‐virally transfected, and then re‐embedded into solid media [9, 10]. However, this compromises the spatial architecture, cellular organization, and tissue polarity of the original organoid, ultimately reverting the system back to a 2D culture. Thus, efficient in situ transfection of organoids in their native 3D state remains challenging.

**FIGURE 1:**
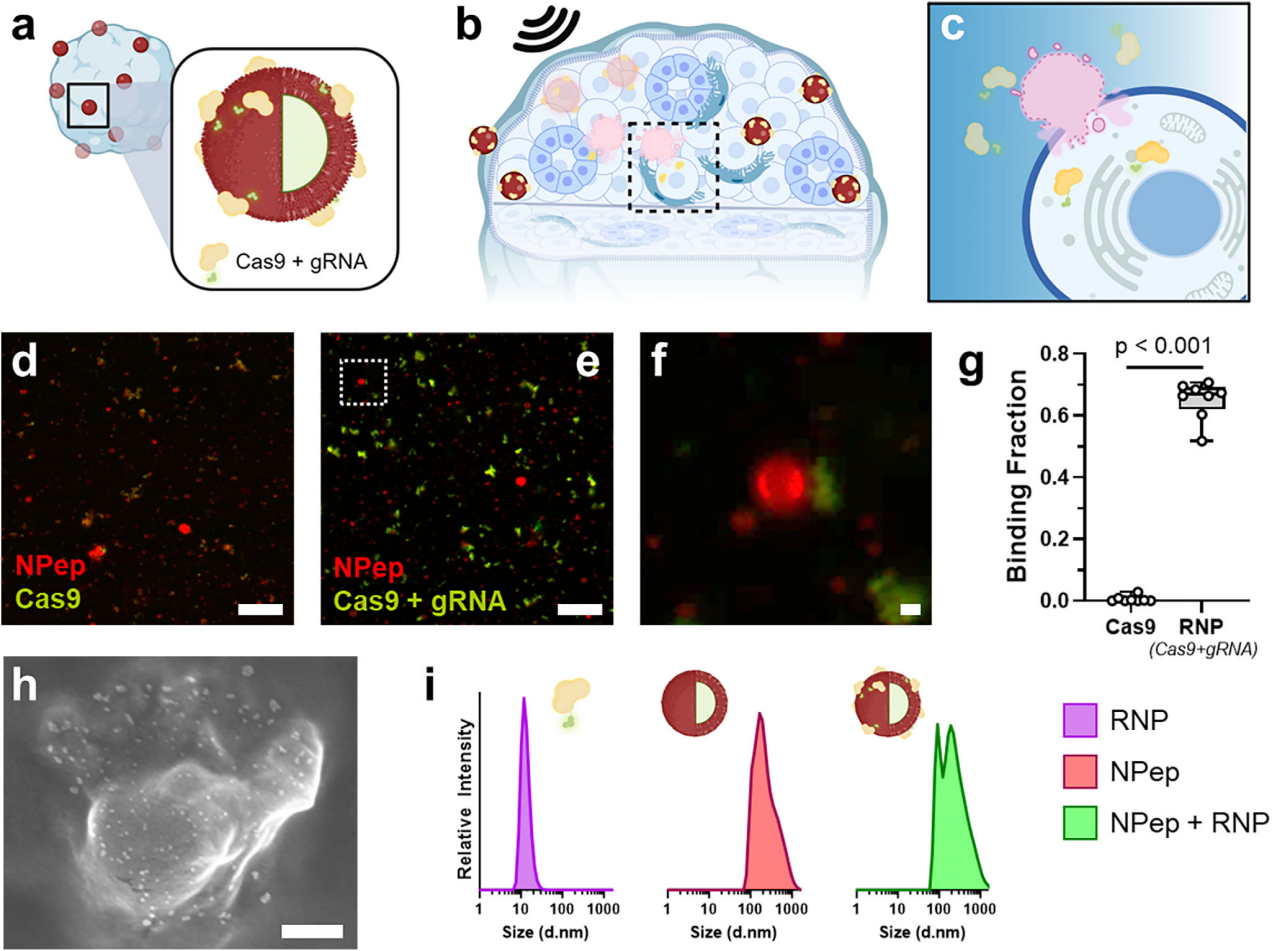
Fabrication and characterization of RNP‐loaded NPep emulsions. (a–c) Schematic of NPep‐mediated acoustic transfection. (a) NPeps are assembled via emulsification of a de novo designed peptide surfactant (red) and perfluoropentane oil (PFP, green). Ribonucleoprotein (RNP) complexes (yellow) electrostatically adsorb to the emulsion surface. (b) RNP‐loaded NPeps diffuse within the organoid interstitium, where they are vaporized via ultrasound to mechanically permeabilize the tissue extracellular matrix and (c) ballistically inject RNP cargo into nearby cells via bubble cavitation. Created with BioRender.com. (d, e) Representative micrographs of DiI‐loaded NPep emulsions (red) incubated with (d) GFP‐Cas9 or (e) the complete RNP complex (GFP‐Cas9 + gRNA). Scale bar = 20 µm. (f) Magnified region of interest from panel e (white dashed box) demonstrating adsorption of GFP‐functionalized RNPs (green) to the NPep surface (red). An atypically large NPep particle is shown to aid visualization. Scale bar = 1 µm. (g) Quantification of the fraction of NPep particles bound to free Cas9 or RNP complexes (n = 8 technical replicates). Statistical significance determined using unpaired Student's *t*‐test with *p* value shown. (h) Representative scanning electron micrograph of an NPep emulsion decorated with adsorbed RNPs (high contrast nodules). NPep emulsion appears as a deflated membrane due to dehydration for imaging. Scale bar = 100 nm. (i) Representative particle size distributions of RNPs (purple), free NPeps (red) or RNP‐loaded NPep vector (green).

Here, we develop a nanomaterial enabled, non‐viral, acoustic transfection method that improves the penetration and cellular accumulation of gene editing ribonucleoprotein (RNP) complexes within intact 3D tissues, relative to standard chemical transfection approaches. As an exemplary model, we use human induced pluripotent stem cell (iPSC) derived kidney organoids given the variety and prevalence of genetic diseases that affect the renal system [11, 12, 13, 14, 15, 16]. Intra‐organoid delivery of CRISPR‐Cas9 RNPs is accomplished using a nanoemulsion vector fabricated from a cell‐targeting peptide surfactant that, when emulsified with a perfluorocarbon solvent, forms an ultrasound‐sensitive liquid droplet referred to as a nanopeptisome (NPep, Figure 1a). Under ultrasound (US), NPeps are engineered to undergo a liquid‐to‐gas phase transition of the particle core to generate microbubbles within the organoid interstitium (Figure 1b). Cavitational collapse of the bubble nuclei mechanically permeabilizes the dense organoid extracellular matrix, and simultaneously ballistically delivers adsorbed RNPs across nearby cellular membranes to affect gene editing (Figure 1c). In this work, we demonstrate that NPep acoustic transfection improves the efficiency, reliability and depth of editing compared to standard lipofection approaches in human kidney organoids.

## 2 Development and Validation of Acoustic Transfection Nanovector

To generate the nanoemulsion delivery vector, we stabilized volatile perfluoropentane (PFP, boiling point = 29°C) nanodroplets using the fluoroamphiphilic peptide emulsifier: F_F_F_F_F_F_GGGCCGGKGRGD‐NH_2_ (F_F_: pentafluorophenylalanine; Figure S1). The fluorinated N‐terminus of the peptide promotes its assembly at the PFP‐water interface, while two cysteines within the central glycine‐rich spacer allow for intermolecular disulfide‐crosslinking between adjacent peptides to stabilize the formed particle [17]. A C‐terminal RGD motif is displayed from the surface of the emulsion to enable binding with α_V_β_3_ integrins highly expressed on the surfaces of major nephron cell types (e.g., podocytes, tubules [18, 19]) to promote interstitial accumulation. The formed emulsions are colloidally and thermodynamically stable, persisting for more than a day at physiologic temperature as superheated PFP liquid droplets (Figure S2).

Loading Cas9 to the NPep vector is accomplished via physical mixing to adsorb the protein to the particle surface. While encapsulation and conjugation methods were considered, we elected a simple mixing protocol to form the NPep‐RNP complex to match the methodology familiar to most researchers when using standard lipofection reagents. Fluorescent confocal studies using GFP‐Cas9 fusion proteins demonstrated that binding of guide RNA (gRNA) to the endonuclease was a prerequisite for its adsorption to the NPep particle surface (Figure 1c–g). This is likely mediated by the cationic surface RGD motif, supporting previous findings on the necessity of the polyanionic gRNA for RNP complexation with cationic nanovectors [20, 21]. Electron microscopy and dynamic light scattering studies confirmed that bound RNPs uniformly decorate the surface of the NPep carrier (Figure 1h) and do not significantly alter particle size distribution (Figure 1i), respectively.

**FIGURE 2:**
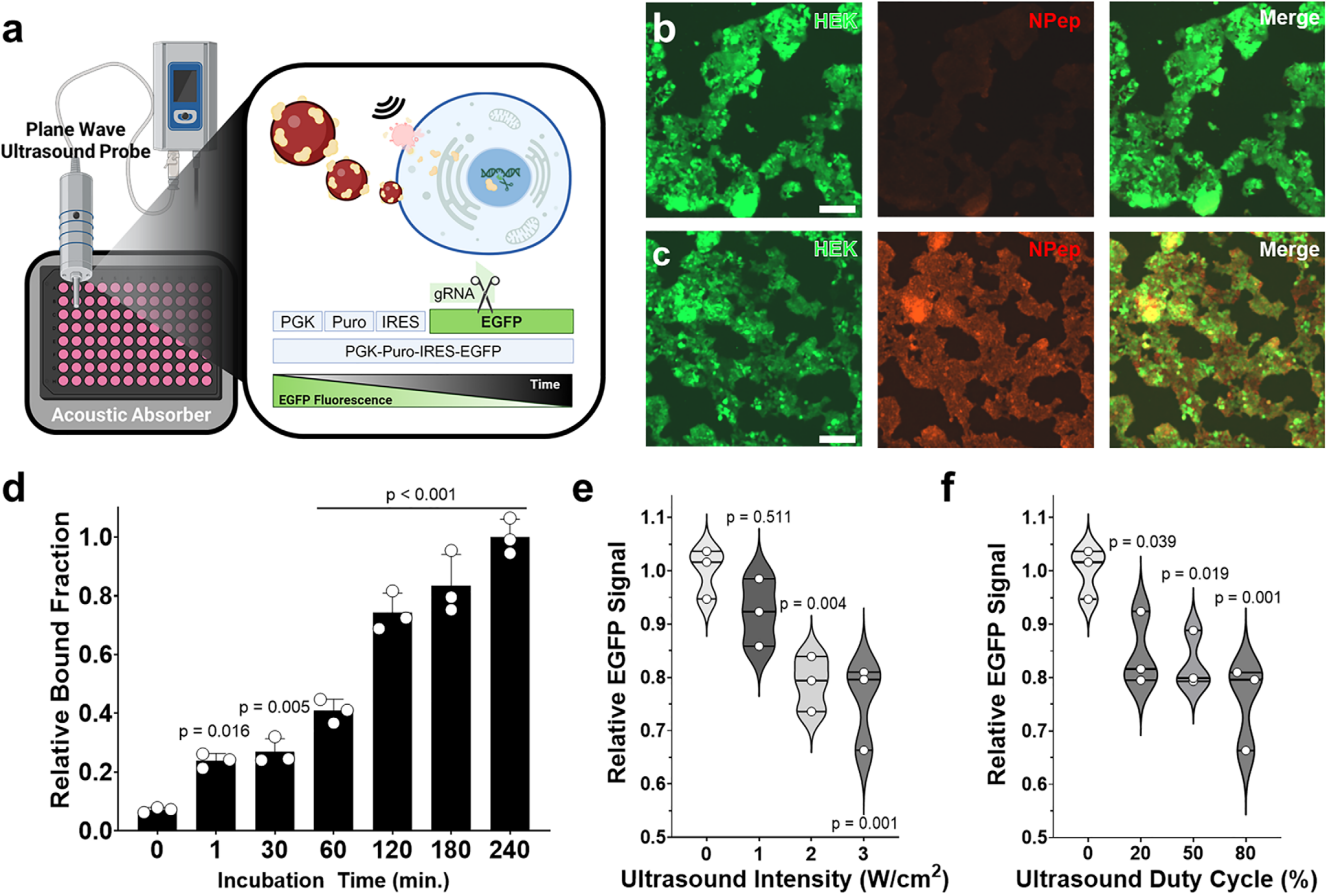
US‐actuated NPep_RNP_ gene editing in 2D culture models. (a) Schematic of the acoustic experimental setup and US‐actuated cavitation of NPeps at the cell surface, leading to intracellular injection of loaded RNPs. Successful delivery of functional RNPs leads to editing of the EGFP gene target and time‐dependent fluorescence knockdown. Created with BioRender.com. (b, c) Representative confocal micrographs of HEK293T‐EGFP (green) and NPep particles (red) at (b) 30 min. and (c) 4 h incubation times. Individual fluorescence channels and merged images are shown for each treatment condition. Scale bar = 200 µm. (d) Relative fraction of cells bound to NPep particles as a function of incubation time. Normalization was performed relative to the maximum average fluorescent signal recorded. (e, f) Relative EGFP signal of HEK293T‐EGFP cells 48 h after treatment with NPep_RNP_ formulations activated at varying acoustic (e) intensities and (f) duty cycles. Data in panels d – f shown as average + s.d., or as a violin plot, of n = 3 technical replicates. All statistical comparisons were made relative to the corresponding ‘0’ condition using unpaired Student's *t*‐test, with *p* values shown. Line in panel d indicates all corresponding conditions have a *p* value less than 0.001 relative to the “0” control.

We next validated the ability of NPeps to acoustically deliver functional RNPs into renal cells, first using 2D culture models. This premise builds upon prior work from our lab, and is corroborated by data shown in Figure S3, demonstrating that vaporization and nonlinear expansion of nanoemulsions under US can ultimately lead to collapse of the formed microbubble; a process referred to as inertial cavitation [17, 22]. A high‐velocity fluid jet formed during asymmetric bubble cavitation penetrates the membrane of emulsion‐bound cells to form a transient pore and, in our system, simultaneously delivers the ejected RNP payload into the cytoplasm (Figure 2a). Here, we validate this acoustic transfection mechanism in the context of CRISPR‐Cas9 gene editors using a knockdown reporter HEK293T‐EGFP human embryonic kidney cell line (see methods, conceptual schematic in Figure 2a, bottom, Figure S4, and Table S1). Importantly, during acoustic transfection, the cavitation vector must be localized to the cell surface to allow the fluidic nano‐jet to impinge upon the plasma membrane. Consequently, we began our investigation by evaluating the time‐dependent localization of fluorescently labeled NPeps to the surface of HEK293T‐EGFP cells. Results in Figure 2b, c show that the particles engage and uniformly decorate the kidney cell surface within a few minutes of exposure, achieving maximum surface density at 4 h of incubation (Figure 2d). Utilizing this optimized incubation time, gene editing of RNP‐loaded NPep formulations, hereafter referred to as NPep_RNP_, was next evaluated as a function of intensity (Figure 2e, Figure S5) and duty cycle (Figure 2f, Figure S6) of the actuating US stimulus. Results show that acoustic intensities ≥2 W/cm^2^ are sufficient to deliver the RNP payload, leading to a statistically significant knockdown of EGFP (Figure 2e). Editing efficiency was additionally found to generally increase as a function of acoustic duty cycle (Figure 2f). Importantly, these US parameters are well below the FDA diagnostic limit of 190 W/cm^2^ spatial peak pulse average intensity [23], demonstrating that this approach can enable US‐guided gene editing without collateral mechanical tissue damage. This is further supported by prior studies from our lab demonstrating that acoustic intensities ≤3 W/cm^2^ are generally well tolerated by a variety of cell types [17, 22, 24].

## 3 Acoustic Delivery of RNPs Into Kidney Organoids

**FIGURE 3:**
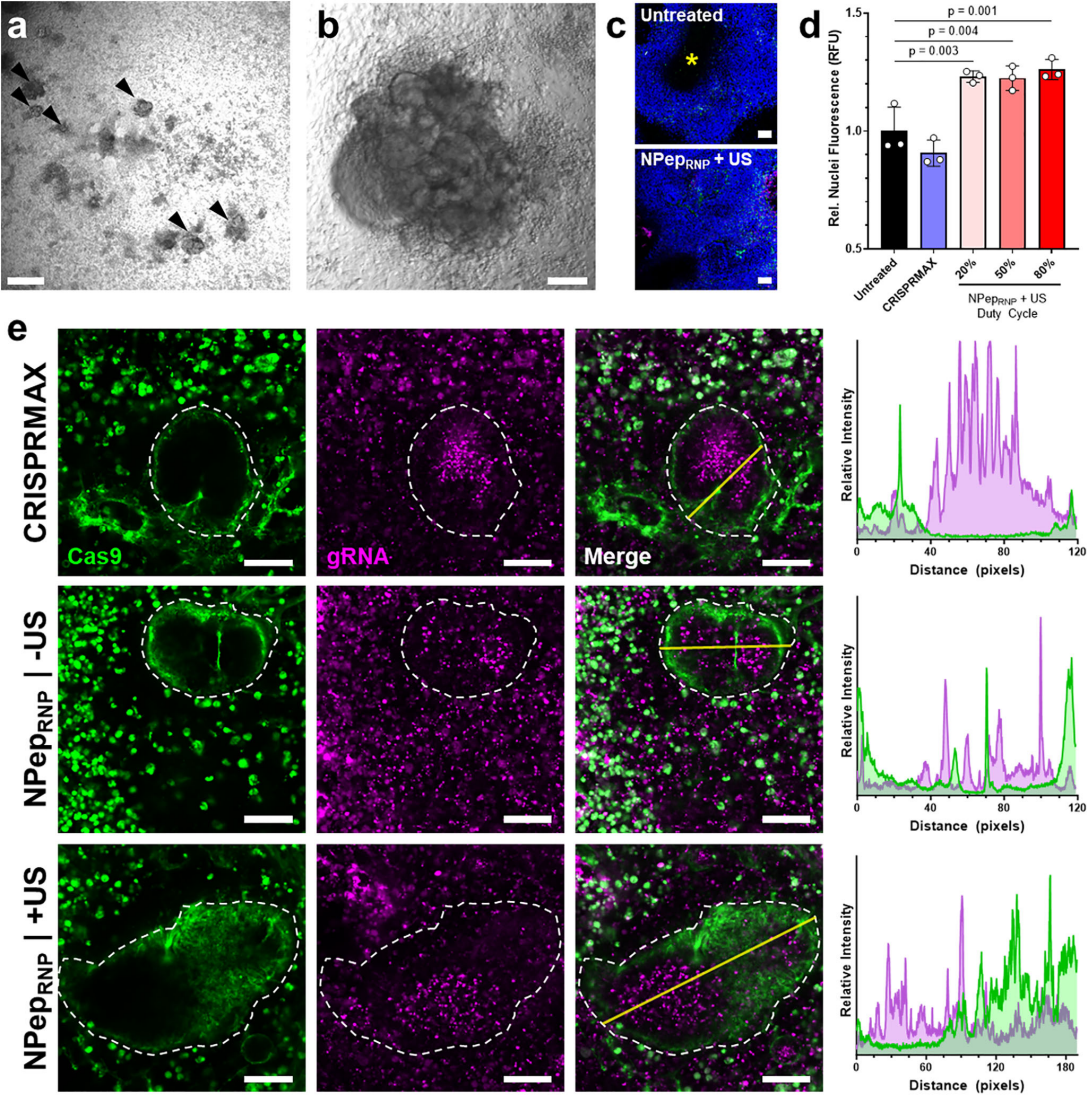
Acoustic delivery of RNPs into 3D kidney organoids. (a, b) Representative brightfield micrographs of a (a) field and (b) single human kidney organoids. Scale bar in panels a and b represent 500 and 100 µm, respectively. Black arrows in panel a denote organoid clusters. (c) Representative fluorescence confocal micrograph of DAPI (blue) stained organoids untreated, or permeabilized with US and NPep_RNP_. Scale bar = 50 µm. Yellow star represents staining deficient organoid core. Sparse green and magenta signal are attributed to Cas9/gRNA as detailed below. (d) Relative nuclei staining in organoid imaging view field (in relative fluorescence units, RFU) in the absence (black) or presence of CRISPRMAX (blue) or NPep_RNP_ vehicles (red) activated at the indicated US duty cycle. Statistical significance determined using unpaired Student's *t*‐test with *p* value shown (n = 3 technical replicates). (e) Confocal micrographs of kidney organoids 24 h after delivery of RNPs prepared from Cas9‐GFP and ATTO 647 gRNA using the indicated vehicle. White dashed line indicates visual estimation of organoid periphery. Right, fluorescence surface overlays of Cas9 (green) and gRNA (pink) signals extracted from the yellow line shown to the left. Scale bars = 100 µm.

Kidney organoids were generated from human iPSCs, as previously described [8, 25], in 96‐well tissue culture plates supported by a Matrigel matrix. The differentiated structures are approximately 100–400 µm in diameter and 40–70 µm in height, containing podocyte, proximal tubule, and distal tubule segments in nephron‐like arrangements (Figure 3a, b). Utilizing this renal model, we first evaluated the ability of NPeps to permeabilize the tissue by studying the diffusion of the nuclei staining dye DAPI within the organoid bulk. Prior work from the Freedman lab [1], and our results in Figure 3c, show that signal deficient regions within the core of DAPI‐stained kidney organoids are a result of poor diffusion of the dye throughout the dense tissue interstitium. Permeabilization of the organoid extracellular matrix (ECM) by US‐activated NPep_RNP_ particles produced more uniform staining throughout the tissue cross‐section (Figure 3c, bottom), improving total nuclei fluorescence by ∼25% relative to untreated and static CRISPRMAX lipofection controls (Figure 3d). However, importantly, this porosification does not result in broad disruption of ECM integrity and micro‐tissue cohesion, as demonstrated through comparative collagen staining (Figure S7).

Next, we evaluated the intra‐organoid delivery of a Cas9‐GFP fusion, and its paired gRNA, before and after US delivery from NPep_RNP_ vectors (Figure 3e). Both the CRISPRMAX control and unactivated particles (NPep_RNP_ | ‐US) showed sequestration of Cas9 at the tissue surface, with limited penetration of the endonuclease into the organoid core. Similarly, both conditions resulted in poor overlap of the delivered Cas9 and gRNA signals across the tissue cross‐section (see fluorescent line plots in Figure 3e, right). US vaporization of NPep_RNP_, and acoustic ejection of the adsorbed RNP complex, led to enhanced intra‐organoid penetration of Cas9, and improved Cas9‐gRNA co‐localization, within the interstitium relative to controls (Figure 3e). These results encouraged further investigation of the editing depth and efficiency of RNPs delivered from the NPep carrier.

**FIGURE 4:**
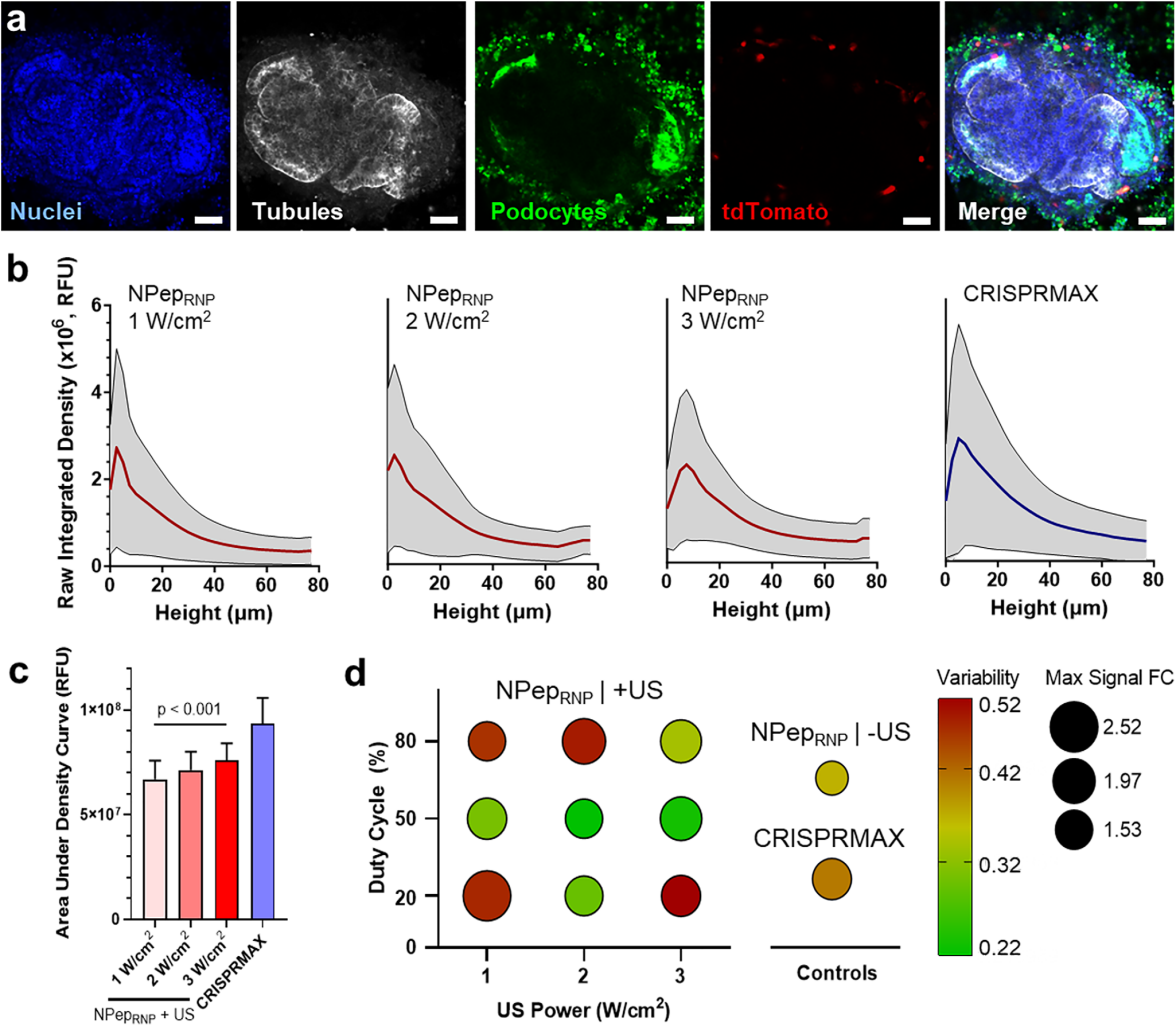
US actuated gene editing of 3D kidney organoids. (a) Representative midsection confocal fluorescent micrograph of a CRISPRMAX edited organoid. Individual channels for cell nuclei (blue), proximal tubules (white), podocytes (green) and tdTomato reporter (red) are shown, along with the merged channel. Scale bars = 100 µm. (**b**) Treatment‐dependent change in relative tdTomato signal (in relative fluorescence units, RFU) as a function of vertical height from the base of the organoid. Treatment condition shown in the upper left corner of each plot (duty cycle = 50%), and results represented as moving average (red or blue line) ± s.d. (grey region). (c) Area under the tdTomato curve from profiles shown in panel b for NPep + US (red) or CRISPRMAX (blue) treated organoids. Statistical comparison for NPep + US treated samples made relative to CRISPRMAX using unpaired Student's *t*‐test, with *p* values shown. (d) Multivariable analysis of max tdTomato signal (circle size) to editing variability (color scale) for kidney organoids edited using NPep_RNP_ formulations activated at the indicated US parameters. Control organoids edited via CRISPRMAX and unactivated NPep_RNP_ (‐US) shown for comparison. Quantitative results are taken from n ≥ 15 technical replicates for each condition, pooled from n ≥ 6 wells across n ≥ 2 biologic replicates.

To evaluate gene editing, we performed transfection using the published calcium shock (CaSH) method on a reporter organoid model derived from a human iPSC line with a fluorescence‐on reporter knocked into the AAVS1 safe harbor locus [25]. Here, successful RNP‐mediated knockout of a stop cassette leads to production of tdTomato. Additional proximal tubule and podocyte markers were included to spatially collate gene editing events to major nephron cell types (Figure 4a). 3D confocal microscopy was then performed to quantify the relative expression of tdTomato as a function of depth of editing within the organoid tissue. Importantly, organoids remained intact for all analyses to preserve positioning of each edited cell. Results in Figure 4b show that treating organoids with RNPs complexed to CRISPRMAX, or NPep_RNP_ activated by US, led to similar peak tdTomato expression levels at ∼20 µm in height from the bottom of the plate surface. We attribute this to the high cell density at the base of the organoid, which adopts a mounded tissue architecture. Measuring the area under the fluorescent curve indicates a monotonic increase in editing efficiency as US intensity is increased from 1 to 3 W/cm^2^ for NPep_RNP_ treated samples (Figure 4c).

Although NPep mediated sonoporation successfully leads to RNP delivery and gene editing, the transfection efficiency of our system was slightly less than that of CRISPRMAX. However, this is counterbalanced by a lower variability in gene editing using NPep vehicles, when stimulated at the optimal US conditions, compared to the CRISPRMAX gold standard (Figure 4d). This is important as, in addition to maximizing editing efficiency, low variance of edits between replicates is a key milestone for reliable transfection technologies. Additionally, we found that editing variability generally decreased with increasing US intensity for NPep_RNP_ treated organoids, with 2 – 3 W/cm^2^ power and 50% duty cycle providing the most reproducible editing results. Ultimately, 3 W/cm^2^, 50% duty cycle, was selected as the optimal US activation condition to maximize editing efficiency and reproducibility.

**FIGURE 5:**
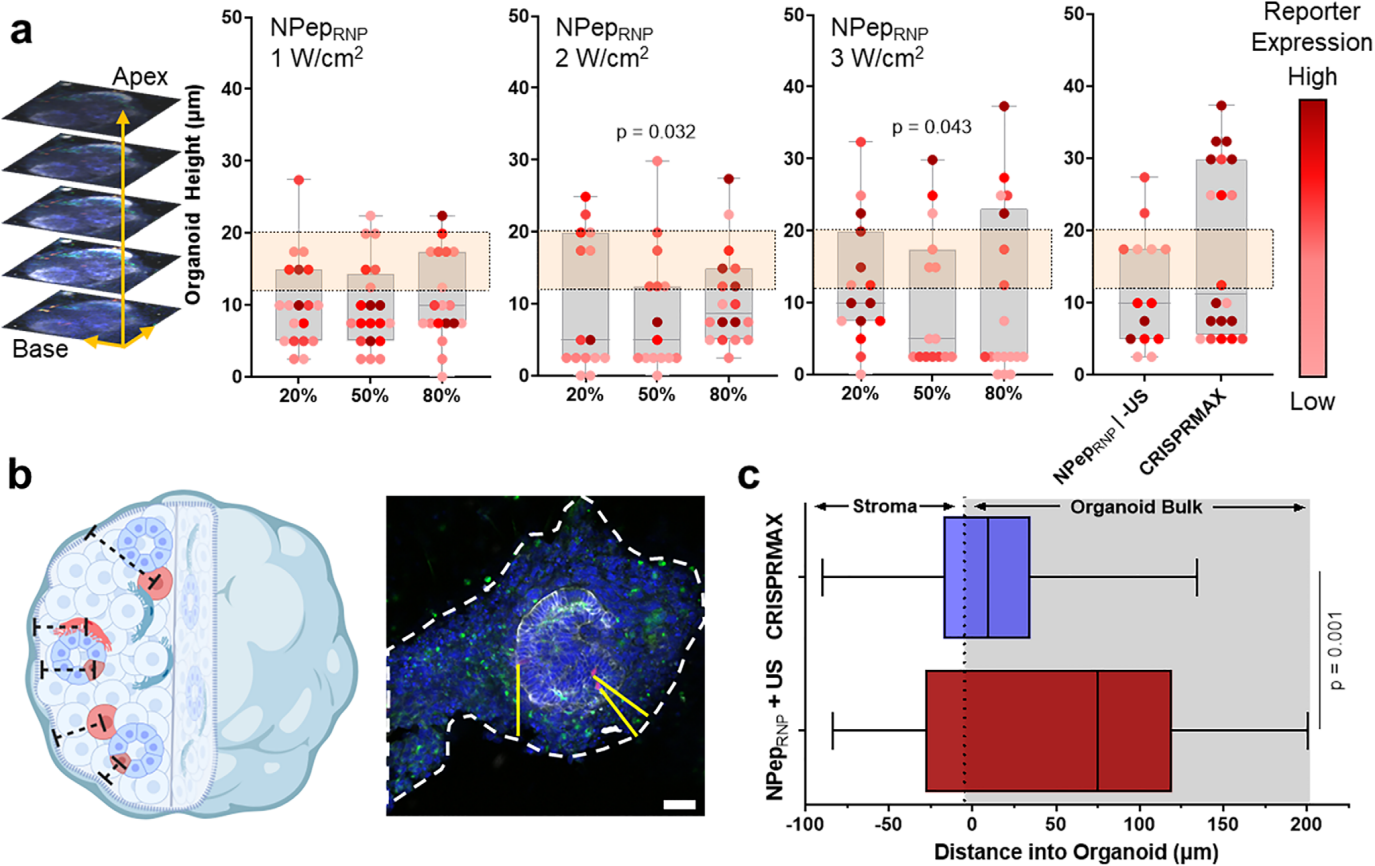
Organoid gene editing depth analysis. (a) Position of maximum tdTomato signal, relative to tissue height from the surface of the plate, within edited kidney organoid sections, as a function of treatment. Color scale represents relative reporter tdTomato fluorescence at signal maximum. Yellow shaded regions signify approximate height range of the organoid core. Statistical significance in panel a determined relative to CRISPRMAX control using one‐way ANOVA (n ≥ 15 technical replicates), with only significant p values (< 0.05) shown for clarity. (b) Schematic (*left*, created with BioRender.com) and exemplary confocal micrograph (*right*) of lateral distance measurement (yellow) of tdTomato positive cells from the organoid peripheral surface (white dashed line) in kidney optical sections. Scale bar = 100 µm. (c) Box‐and‐whisker plot of edited cell distance from organoid surface (dashed line), either into the stroma (unshaded) or organoid bulk (shaded grey region), for CRISPRMAX and NPep_RNP_ + US (3W/cm^2^, 50% DC) treated tissue samples. Statistical significance in panel c determined using unpaired Student's *t*‐test with *p* value shown (n > 18 technical replicates, pooled from n ≥ 2 biologic replicates).

During these studies we observed a marginal shift of the peak maximum tdTomato signal further into the bulk of the organoid when using NPep_RNP_ + US (3 W/cm^2^) treatments relative to CRISPRMAX controls (Figure S8). This motivated us to systematically interrogate the depth of editing in organoids between the static (CRISPRMAX, NPep_RNP_ without US activation) and active (US vaporized NPep_RNP_) transfection conditions. Comparisons reported in Figure 5a were obtained from digital sections of 3D confocal image stacks to obtain individualized z‐stack plots with minimal accessory signal, and then plotting the height at which the maximum tdTomato signal was measured. Each organoid is therefore represented as an individual point, collated with the total tdTomato reporter expression (color scale) to assess how treatment condition and US parameters alter the depth of editing within the organoid bulk (Figure 5a). This analysis showed that, in general, increasing US intensity and duty cycle led to a higher incidence of editing in the NPep_RNP_ treated organoid core (∼12 – 20 µm height), where proximal tubule density is the highest. Conversely, CRISPRMAX editing generally occurs within the underlying basal stromal layer, or at the surface of the structures. Taken together, our data suggest that acoustic actuation of the NPep_RNP_ particle mechanically drives the adsorbed RNPs into the organoid bulk, enabling improved editing of internal cells that are otherwise generally inaccessible to passive transfection technologies.

In addition to this vertical (*z*‐axis) editing analysis, we also compared the lateral depth (*x/y* axis) of editing within the organoid sections (Figure 5b). Our attention focused specifically on comparing CRISPRMAX lipofection to the best observed NPepRNP + US parameters in terms of replicability (Figure 4d) and depth of editing (Figure 5a). For this analysis, the centroids of all tdTomato expressing cells in each z‐stack slice were located, and their individual distances from the organoid surface measured (see exemplary image in Figure 5b). This analysis demonstrates that CRISPRMAX mediated transfection is limited largely to the exterior stroma and organoid surface, while acoustically actuated NPep_RNP_ treatment edits cells significantly deeper within the tissue bulk (Figure 5c). These statistical findings are supported in 3D image reconstructions, with representative confocal micrographs shown in Figure S9. In sum, these studies suggest that mechanical permeation of NPeps into the tissue interstitium under US, and ballistic ejection of loaded RNPs from the cavitating particle surface, promotes improved editing of cells within the interior of dense tissues relative to static lipofection reagents.

## 4 Cell Editing Specificity

**FIGURE 6:**
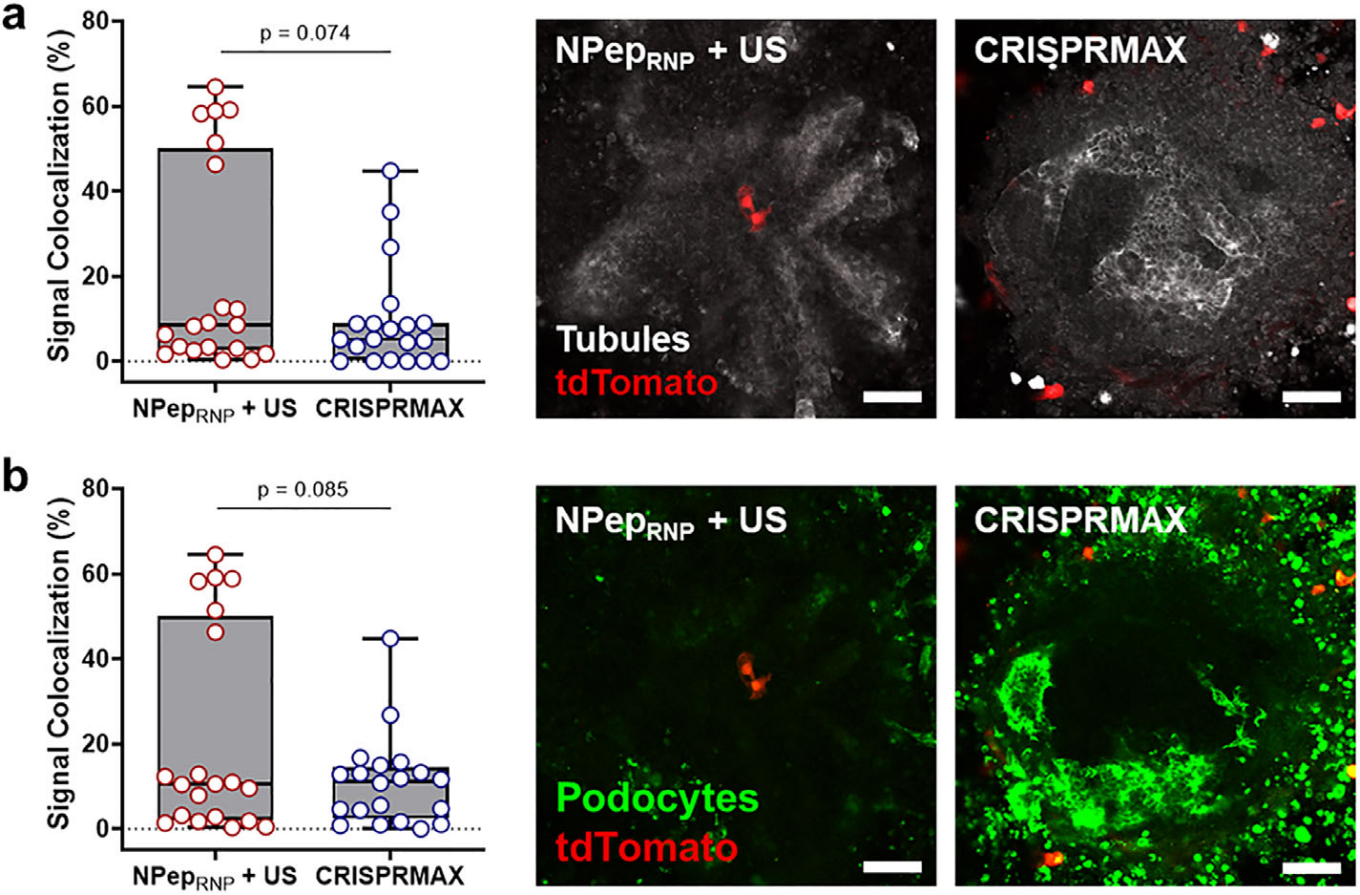
Phenotypic specificity of NPep_RNP_ gene editing in kidney organoids. (a, b) *Left*: Percent colocalized voxel volume above threshold fluorescence of the tdTomato signal (red) with either (a) proximal tubule (LTL, white) or (b) podocyte (podocalyxin, green) phenotypic markers, following treatment with NPep_RNP_ + US (3 W/cm^2^, 50% duty cycle) or CRISPRMAX. Each point represents an individual organoid. Statistical significance determined using unpaired Student's *t*‐test, with *p* values shown (n = 21 technical replicates across n = 3 biologic replicates). Right: Representative images of stained organoid sections. Scale bars = 100 µm.

To investigate cell‐specific editing in organoids treated with CRISPRMAX and NPep_RNP_, we evaluated colocalization of the tdTomato reporter signal with proximal tubule and podocyte phenotypic markers (Figure 6a, b). While a general trend of greater editing within both tubules and podocytes for NPep_RNP_ treated organoids was observed, relative to the CRISPRMAX controls, these correlations were not statistically significant (*p* > 0.05). This may simply be due to the tendency for these nephron cell types to be localized closer to the interior of the organoid, where NPep delivered RNPs gain better access over static CRISPRMAX lipofection. Additionally, the apparent nephron editing bias for NPep_RNP_ may also be due, in part, to binding of the vector's surface displayed RGD motif with integrins highly expressed on podocytes and tubules [18, 19]. However, additional cell binding studies did not demonstrate a significant difference between RGD‐targeted NPep formulations and binding‐deficient control particles (Figure S10). Taken together, our data suggests that ballistic ejection of RNPs from the NPep carrier leads to successful editing of nephron cells deep within the organoid bulk, rather than the collateral mesenchymal layer or superficial surface strata.

## 5 Outlook

A key technologic hurdle for the clinical translation of CRISPR‐Cas9 based gene editing tools is precise control of RNP delivery to sites deep within target solid tissues and organoids. This has been traditionally difficult to achieve using standard, non‐viral, transfection techniques that are unguided and rely on passive interactions with target cells, leading to editing that is largely limited to the tissue periphery. US actuated NPep emulsions are capable of on‐demand and interstitial delivery of CRISPR‐Cas9 RNPs through local sonoporation effects generated during particle acoustic cavitation. Because this behavior can be controlled using an exogenous US pulse, transfection can be potentially spatiotemporally guided. Further, exploiting the microbubbles that result from NPep US vaporization as contrast‐enhancing nuclei can enable imaging‐based guidance of delivery in future applications. In principle, such an approach is compatible with a broad range of base editing proteins, as well as RNA and DNA, to enable tissue‐specific gene repair. Further, the peptide surfactant, and specifically the targeting ligand, can be altered and optimized to engender cell‐specific editing fidelity. Finally, rational modification of NPep size, and the boiling point of the perfluorocarbon particle core, can also allow for further control over the acoustic response of the delivery vector. Thus, NPeps represent a physicochemically versatile RNP delivery platform that can be tailored for a variety of biomedical applications.

Given our demonstration of editing in kidney organoids, and the prevalence of genetic mutations in kidney disease, our attention turns toward utilizing this technology to enable clinically meaningful genetic therapy of renal disease. For example, polycystic kidney disease (PKD) is a genetic disorder frequently caused by pathogenic variants in *PKD1* or *PKD2* for which a phenotypic organoid model exists [8], making it an attractive candidate for US‐guided editing using our platform. Conveniently, α_V_β_3_ integrin is overexpressed in renal cystic cells [19], making this a potential target of our current RGD‐targeted NPep formulation. While the large size of NPeps may present a barrier to kidney delivery, prior research suggests that compliant and deformable particles, like the NPep emulsions described here, can successfully pass through the glomerulus [26]. Thus, our platform not only has the potential to advance the study of renal disease in organoid and in vivo models, but is poised to advance clinically relevant therapeutic gene editing strategies that can impact a variety of genetic diseases in kidney tissue, and beyond. That said, preclinical translation of our technology, although an improvement over established non‐viral approaches, will require further optimization of the carrier stability, acoustic sensitivity and gene editing efficiency; which remain the focus of on‐going work.

## 6 Materials and Methods

### 6.1 Materials

12‐well plates, Dulbecco's Modified Eagle's Medium (DMEM) with L‐glutamine (10‐013‐CV), phosphate buffered saline (PBS, 1x) without calcium and magnesium, fetal bovine serum (FBS), and trypan blue solution (0.4% w/v) were purchased from Corning. *N,N*‐Diisopropylcarbodiimide (DIC), trifluoroacetic acid (TFA), piperidine, Fmoc‐pentafluoro‐phenylalanine (Fmoc‐F_F_) were purchased from Chem‐Impex Rink amide ProTide resin (LL) (100‐200 mesh), oxyma pure, and all Fmoc‐protected amino acids, except Fmoc‐F_F_, were purchased from CEM. *N,N*‐Dimethylformamide (DMF), thioanisole, and dimethylsulfoxide (DMSO) were purchased from Alfa Aesar. 1,2 ethanedithiol was purchased from Acros Organics. Anisole was purchased from TCI Chemicals. Diethyl ether, formic acid, 1,1'‐Dioctadecyl‐3,3,3',3'‐Tetramethylindocarbocyanine Perchlorate (DiI), Slide‐A‐Lyzer 3.5 kDa MWCO dialysis cassettes, 96‐well tissue culture plates, 96‐well black‐walled glass bottom tissue culture plates, calcium phosphate, puromycin dihydrochloride, hygromycin B (50 mg/mL), blasticidin S HCl (10 mg/mL), penicillin‐streptomycin (pen‐strep, 10 000 U/mL), Advanced RPMI, GlutaMAX supplement, B‐27 supplement, Opti‐MEM Reduced Serum Media, Lipofectamine CRISPRMAX, paraformaldehyde (16%, w/v aqueous solution), Triton X‐100 Surfact‐Amps Detergent Solution, Streptavidin (Alexa Fluor 647 conjugate) and Donkey anti‐goat IgG (H+L) cross‐adsorbed secondary antibody (Alexa Fluor 488) were purchased from Thermo Fisher Scientific. Perfluoropentane (PFP) was purchased from SynQuest Laboratories. pLVK PGK Puro‐IRES‐EGFP was purchased from Takara Bio Inc. Plasmids for HEK293T knockout validation were purchased from Addgene. Alt‐R S. p. Cas9 Nuclease V3, Alt‐R S. p. Cas9‐GFP V3, Alt‐R CRISPR‐Cas9 Negative control crRNA #1, Alt‐R CRISPR‐Cas9 tracrRNA, Alt‐R CRISPR‐Cas9 tracrRNA ATTO 647, and Cas9 dilution buffer were purchased from Integrated DNA Technologies. 12.7 mm aluminum specimen mounts were purchased from Ted Pella Inc. mTeSR1 media and Accutase were purchased from STEMCELL Technologies. CHIR99021 was purchased from Tocris Bioscience. Paraformaldehyde 4% was purchased from Santa Cruz Biotechnology. Millipore 0.22 and 0.45 µm pore membrane syringe filters and 4‐(2‐hydroxyethyl)‐1‐piperazineethanesulfonic acid (HEPES, 1 m) were purchased from Millipore‐Sigma. Lotus Tetragonolobus Lectin (LTL, biotinylated) was purchased from Vector Laboratories. Human podocalyxin antibody was purchased from R&D Systems.

### 6.2 Peptide Synthesis

The peptide emulsifier for NPep (F_F_F_F_F_F_GGGCCGGKGRGD‐NH_2_) was synthesized and purified by Fmoc‐based solid‐phase peptide chemistry using a CEM Liberty Blue microwave peptide synthesizer with DIC/oxyma activation, and Fmoc deprotection by 20% piperidine in DMF. Peptide cleavage from ProTide Resin and side chain deprotection were achieved by treatment with a cleavage cocktail (TFA/thioanisole/1,2 ethanedithiol/anisole = 90:5:3:2) for 3 h under argon. Resin was filtered and washed on a ceramic frit. Crude peptide was precipitated from cleavage cocktail by adding cold diethyl ether to the solution, then the product was dried via overnight lyophilization. Peptide was purified by reverse phase HPLC (Shimadzu) with Phenomenex semi‐prep Luna C18 column using a gradient of 0%–25% standard solvent B (90% acetonitrile, 10% water, 0.1% TFA) over 10 min, followed by 25–60% standard solvent B over 35 min, then standard solvent B to 60 min. Standard solvent A was 0.1% TFA in water for HPLC. The purified peptide was identified by LC‐MS (Shimadzu, LC‐MS 2020) equipped with Phenomenex C18 analytical column using a gradient of 1% standard solvent B (90% acetonitrile, 10% water, 0.1% formic acid) per minute for 100 min. 0.1% formic acid in water was standard solvent A for LC‐MS.

### 6.3 NPep Synthesis

Lyophilized pure peptide was reconstituted in PFP, vortex mixed and sonicated. NPep formation was initiated by adding the peptide/PFP mixture 1:15 to cold DI water, spinning at 1500 rpm. The emulsion was stirred for 1 h before the full volume was transferred to a Slide‐A‐Lyzer 3.5 kDa MWCO dialysis cassette. NPeps were dialyzed overnight against 2.5% DMSO to promote disulfide crosslinking for NPep stability. Dialysis solution was exchanged for pure DI water 1 h before removing nanoemulsions from cassettes for use. Particle size and relative frequency were estimated using dynamic light scattering (DLS) on a Malvern ZetaSizer at the Materials Research Institute at The Pennsylvania State University. To validate acoustic droplet vaporization, brightfield confocal microscopy was performed before and after exposure to ultrasound at 0 – 2 W/cm^2^. FIJI software was used for imaging based size estimation. To evaluate RNP adhesion to NPep, DiI loaded NPeps were also synthesized. DiI was dissolved in HFIP, then added 1:3 to PFP. Peptide was dissolved in the HFIP/PFP solution, then used as described for NPep synthesis.

NPep particle concentrations were standardized before each replicate experiment via optical density measurements (OD_600_), with correlation of OD_600_ values to particle concentration via a calibration curve (Figure S11). A typical concentration range of NPeps used in experiments is ∼20 000 – 118 000 particles/µL. To prepare the calibration curve, DiI‐loaded emulsions were diluted in water to an OD_600_ = 1 and a series of dilutions (n = 3 for each) were made from this suspension. The OD_600_ value for all the samples was measured and further diluted 1:20 in ultrapure water to determine the particle count in a 50 µl volume.

### 6.4 HEK293T EGFP Cell Line Production and Binding Assays

HEK293T (ATCC RRID: CVCL_0063, 2023) cells were confirmed to be contamination free, and upon receipt modified to constitutively express EGFP by lentiviral transfection. EGFP lentivirus was made by transfection of 6 × 10^5^ HEK293T cells with 5 µg of pLVK PGK Puro‐IRES‐EGFP plasmid and 1 µg of each lentiviral helper plasmid (TAT, gag‐pol, vsvg, rev) using calcium phosphate, as previously described [27]. Media was replenished with fresh DMEM (10‐013‐CV, Corning) 24 h after transfection. Lentiviral media was syringe filtered 24 h later using a 0.45 µm filter and added dropwise to fresh HEK293T cells. After 3 d infection, HEK293T cells were selected with 2.5 µg/mL puromycin until >95% of the live cells were GFP+ by BD Accuri C6 flow cytometer. Knock out validation of selected sgRNA was accomplished by Sniper Cas9 lentiviral transfection with rationally selected controls and target sites for gRNA (Figure S4 and Table S1). The same procedure was used for lentiviral delivery of Sniper Cas9 [28] (Addgene #138559, 5 µg/mL blasticidin) and gRNA cassettes [29] (Addgene #104991, 200 µg/mL hygromycin). Cells were maintained in DMEM with L‐glutamine, supplemented with 10% FBS and 1X (100 U/mL) pen‐strep. Populations were re‐challenged with selection antibiotics puromycin when loss of signal was observed, but passaged into normal media >24 h before experiments.

For cellular binding assays, HEK293T‐EGFP cells were seeded at 10 000 cells/cm^2^ then cultured overnight. For cell binding experiments, DiI‐loaded NPeps, prepared from either RGD or RGE containing peptide surfactants, were introduced to cells (10% final volume) in complete DMEM 0–4 h before washing twice in PBS and imaging as described below by fluorescent microscopy. Bound fraction was estimated by area fraction of DiI/EGFP positive cells.

### 6.5 RNP Preparation

Cas9 ribonucleoprotein complexes were formed before introduction to sample wells. A mix of reporter cassette‐targeted sgRNAs were prepared as previously described. IDT tracrRNA and Negative Control crRNA #1 were annealed at 95°C for 5 min to create negative control duplex sgRNA. For testing delivery of fluorophore‐tagged RNP, ATTO 647 tracrRNA was annealed with the same Negative Control crRNA #1. Cas9 stock was diluted to working concentration in 0.22 µm‐filtered Cas9 working buffer (KCl pH 7.5/HEPES/dH_2_O 15:2:83). Excess sgRNA was incubated with Cas9 at room temperature for 15 min to allow RNP complexation, then used immediately for all studies.

### 6.6 Confocal Fluorescent Microscopy

NPep adhesion to and EGFP knockout in HEK293T EGFP were observed under fluorescent microscopy using a Biotek Cytation 3 Imaging Plate Reader. Samples were imaged in brightfield, Texas Red (λ_ex_ = 586 nm, λ_em_ = 647 nm), GFP (λ_ex_ = 469 nm, λ_em_ = 525 nm), and DAPI (λ_ex_ = 380 nm, λ_em_ = 435 nm). Kidney organoids were imaged in brightfield channel to qualitatively track organoid health during culture.

RNP adhesion to DiI NPep and kidney organoid immunofluorescence were imaged using a Zeiss LSM 880 confocal microscope with AiryScan Fast at the Huck Institutes of Life Sciences Microscopy at The Pennsylvania State University. Channel selection was optimized for low laser crosstalk using Zeiss Zen Black imaging software. For RNP adhesion to NPep, images were captured in two channels: GFP (λ_ex_ = 488 nm, λ_em_ = 493–597 nm) and DiI (λ_ex_ = 541 nm, λ_em_ = 638–759 nm). Kidney organoids were imaged in four channels: DAPI (λ_ex_ = 405 nm, λ_em_ = 410–483 nm), AlexaFluor 488 labeled Podocalyxin (λ_ex_ = 488 nm, λ_em_ = 503 – 560 nm), tdTomato (λ_ex_ = 541 nm, λ_em_ = 566–628 nm), ATTO 647 labeled Streptavidin (λ_ex_ = 633 nm, λ_em_ = 648‐ 700 nm).

### 6.7 Kidney Organoid Differentiation

Human kidney organoids were differentiated from *AAVS1* ^LSL‐tdTom^ iPS cells (WIBR3, RRID:CVCL_9767 Hockemeyer lab, UC Berkeley) in 3D culture as previously described [25]. All lines were tested and found to be negative for contamination (Hockemeyer laboratory) [30]. Organoids were treated as described below on d21 after seeding, then further maintained until d28.

### 6.8 Transfection Assays

Organoid transfections were performed using calcium shock (CaSh) conditions to enhance transfection [25]. Lipofection was accomplished by mixing Cas9 and gRNA 5:6 in DMEM (for cell studies, 10‐013‐CV, Corning) or Calcium Shock (CaSh) transfection media (for organoid studies. Calcium‐free DMEM 1X (high glucose, no glutamine, no calcium, 21068‐028, Gibco) supplemented with 1X GlutaMAX (35050061, Thermo Fisher), 1X Sodium Pyruvate 10mM (11360‐070, Gibco), 10 ng/ml Recombinant Human FGF‐basic (100‐18B, Peprotech)) to a final theoretical concentration of 20 µM RNP in media. CRISPR‐Plus reagent was added to the RNP media solution to complete mixture A. Mixture B was formed by CRISPRMAX reagent and DMEM or CaSh transfection media [25]. Mixture B was immediately added to mixture A and mixed. Then, the final mixture was added 3:2 to DMEM or CaSh transfection media in wells. After plates incubated at 37°C, 5% CO_2_ for 2.5 h, 100 µL Opti‐MEM was added to each well, then plates continued incubation. After 24 h incubation, media was exchanged for DMEM (for cell studies) or RB (for organoid studies) media [25].

NPep treatment media was made by adding NPeps to 10% concentration in DMEM or CaSh transfection media, for HEK293T cells and organoids, respectively. 70 µL NPep treatment media was added to each treatment well in 96‐well plates. Plates incubated 4 h at 37°C, 5% CO_2_ to allow for NPep adhesion to cells. Cas9 was gently mixed 5:6 with sgRNA in DMEM or CaSh transfection media to a final theoretical concentration of 20 µm RNP in media. 32.6 µL RNP‐containing media was added to each treatment well. Wells were sonicated using a 1 MHz, 6 mm tip transducer on a Nepagene Sonitron GS at variable intensity and duty cycle. Intensity varied from 0–3 W/cm^2^, while duty cycle varied from 0%–80% in 10 ms intervals. Exposure time remained constant at 90 s per well. Transducer tip was sterilized and submerged in growth media to expose samples directly to US mechanical waves. After 2.5 h, 100 µL Opti‐MEM was added to each well. Plate was incubated at 37°C, 5% CO_2_ before exchanging media for DMEM or RB media.

### 6.9 Immunofluorescence

Human kidney organoids were maintained 7 d after treatment, refreshing media every 2 d, before fixing for imaging. Kidney organoids were fixed in paraformaldehyde by adding 8% paraformaldehyde 1:1 to wells containing RB media. Organoids were rinsed with 1X PBS before adding blocking buffer (5% FBS, 0.3% Triton X‐100 in 1X PBS). Samples were blocked for 60 min. Biotinylated Lotus Tetragonolobus Lectin (LTL) and Goat anti‐Human Podocalyxin antibodies were diluted to working concentration (5 and 0.5 µg/mL, respectively) in Antibody Dilution Buffer (10 mg/mL BSA, 0.3% Triton X‐100 in 1X PBS). Blocking buffer was removed and replaced with dilute primary antibody solution. Samples were incubated with primary antibodies overnight at 4°C. Secondary stains (Streptavidin and Donkey anti‐goat IgG) were prepared in Antibody Dilution Buffer to working concentration (4 µg/mL each). Samples were rinsed and secondary antibodies, along with DAPI nuclear stain, were introduced before incubating overnight at 4°C. Samples were rinsed and stored in 1X PBS before imaging.

To evaluate editing efficiency, entire wells were exposed to their defined treatment condition. Two or three organoids were randomly selected within each well for analysis, and 3D confocal micrographs were captured to quantify the relative expression of tdTomato as a function of depth within the organoid tissue. Organoids were further digitally sectioned to remove signal from stromal cells outside the 3D organoid structure, where defined.

### 6.10 Scanning Electron Microscopy

Scanning electron microscopy (SEM) was accomplished by drying samples (10 µL) of nanoemulsions with Cas9 RNP on 12.7 mm Aluminum specimen mounts. Samples were gold‐palladium coated using a Bal‐tec SCD‐050 sputter coater. Micrographs were obtained using a Zeiss VP‐FESEM with electron high tension of 5 kV and working distance 3.3 mm. Facilities used for SEM were courtesy of the Huck Institutes of Life Sciences Microscopy Facility at The Pennsylvania State University.

### 6.11 Image and Statistical Analysis

Images were analyzed using ImageJ with FIJI plug‐in toolkit. Organoids within images were digitally sectioned manually based on nuclei, podocalyxin and LTL signal for editing efficiency and penetration depth analyses. To assess organoid editing depth in x/y, the organoid area and position defined by digital sectioning and measured position of each cell were used to estimate each cell's distance into the organoid. Percent signal colocalization was measured to assess relationship between reporter and phenotype signals. Unless otherwise specified, data represents mean ± standard deviation of n = 2 – 3 biologic replicates, within each n = 3 technical replicates per experiment. One‐way ANOVA or Student's *t* test (two‐tailed, unpaired) with confidence intervals were used to determine significance where described, calculated in GraphPad Prism 10.

## Conflicts of Interest

B.S.F. is an inventor on patents including “3D differentiation of epiblast spheroids into kidney tubular organoids modeling human microphysiology, toxicology, and morphogenesis” and “High‐throughput automation of organoids for identifying therapeutic strategies”. B.S.F. holds an ownership interest in Plurexa LLC. Plurexa was not involved in the published research, and none of the preceding interests affected in any way the results of the paper.

## Supporting information




**Supporting File**: advs75998‐sup‐0001‐SuppMat.docx.

## Data Availability

The data that support the findings of this study are available in the supplementary material of this article.
