# Supplementary material for: Ultrasound‐Actuated Gene Editing in Human Kidney Organoids
Source: Adv Sci (Weinh). 2026 Jun 15;13(49):e20402. doi: 10.1002/advs.202520402 (PMC13336553; doi:10.1002/advs.202520402)
Supplement: Supplementary file 1 [file ADVS-13-e20402-s001.docx]

**SUPPORTING INFORMATION**

**Ultrasound-Actuated Gene Editing in Human Kidney Organoids**

Michael A. Miller^1^, Nicole Vo^2,3,4,5^, Utkarsh^1^, Ivan Sokirniy^6,7^, Justin Pritchard^1,7^, Benjamin S. Freedman^2,3,4,5,8,9,10^, Scott Medina^1,6,7,11*^

*^1^Department of Biomedical Engineering, Pennsylvania State University, University Park, PA 16802, USA*

*^2^Division of Nephrology, University of Washington School of Medicine, Seattle, WA 98109, USA*

*^3^Kidney Research Institute, University of Washington School of Medicine, Seattle, WA 98109, USA*

*^4^Institute for Stem Cell and Regenerative Medicine, University of Washington School of Medicine, Seattle, WA 98109, USA*

*^5^Department of Medicine, University of Washington School of Medicine, Seattle, WA 98109, USA*

*^6^Molecular, Cellular, and Integrative Biosciences Program, Pennsylvania State University, University Park, PA 16802, USA*

*^7^Huck Institutes of the Life Sciences, Pennsylvania State University, University Park, PA, USA,*

*^8^Department of Laboratory Medicine & Pathology, University of Washington School of Medicine, Seattle, WA 98109, USA*

*^9^Department of Bioengineering, University of Washington, Seattle, WA 98109, USA*

*^10^Plurexa LLC, Seattle, WA 98109, USA*

*^11^Center for Biodevices, Pennsylvania State University, University Park, PA 16802, USA*

**Contents**

[Figure S1.	3](#_Toc209087722)

[Figure S2.	4](#_Toc209087723)

[Table S1.	5](#_Toc209087724)

[Figure S3.	6](#_Toc209087725)

[Figure S4.	7](#_Toc209087726)

[Figure S5.	8](#_Toc209087727)

[Figure S6.	9](#_Toc209087728)

[Figure S7.	10](#_Toc209087728)

[Figure S8.	11](#_Toc209087728)

[Figure S9.	12](#_Toc209087728)

[Figure S10.	13](#_Toc209087728)

[Figure S11.	14](#_Toc209087728)

Figure S1. (**a**) Molecular structure of F_F_F_F_F_F_GGGCCGGKGRGD-NH_2_, and its (**b**) HPLC trace, and (**c**) ESI-MS mass spectrum.

Figure S2. Colloidal and thermodynamic stability, as determined via optical density measurements (OD_600_), of NPep particles incubated in DMEM culture media over 0 – 24 hours at 4 – 37°C. Statistical significance determined via two-way ANOVA, with only significant correlations (p < 0.05) shown for clarity.

Figure S3. Ultrasonic vaporization of NPep emulsions. (**a**) Triplicate representative brightfield microscopy images of NPep particles before (-US) and after (+US) acoustically mediated phase transition of liquid emulsions to gaseous microbubbles. Scale bar = 100 μm. (**b**, **c**) Quantification (b) and histographic representation (c) of particle sizes of NPeps before (-US) and after (+US) acoustic vaporization. Statistical significance in panel b determined via Student’s t-test, with p value shown.

Figure S4. (**a**) EGFP gene insert by lentiviral transduction with gene knockout candidate locations. (**b**) percent EGFP positive HEK293T-EGFP cells after lentiviral knockout by three knockout candidates (gEGFP_KO1, gEGFP_KO2, gEGFP_KO3), an untargeted control (gNT), and an AAVS1 safe harbor control (gAAVS1). 0% expression control indicated by No EGFP condition.

Table S1. gRNA sequences for HETK293T-EGFP knockout selection

| **gRNA** | **Sequence** |
| --- | --- |
| gAAVS1 | GGGGCCACTAGGGACAGGAT |
| gNT | ACGGAGGCTAAGCGTCGCAA |
| EGFP_KO1 | GGGCGAGGAGCTGTTCACCG |
| EGFP_KO2 | GAAGTTCGAGGGCGACACCC |
| EGFP_KO3 | GAGCTGGACGGCGACGTAAA |

Figure S5. Representative images of HEK293T-EGFP after knockout via NPep_RNP_ treatment as a function of US intensity (1 MHz, 80% duty cycle, 90 sec. exposure time). Scale bar = 2 mm.

Figure S6. Representative images of HEK293T-EGFP after knockout via NPep_RNP_ treatment at 0 or 3 W/cm^2^ US exposure as a function of acoustic duty cycle (1 MHz, 90 sec. exposure time). Scale bar = 2 mm.

Figure S7. Representative fluorescent confocal micrographs (**a**) and signal quantification of collagen staining (**b**) within organoids treated in the absence (untreated) or presence of CRISPRMAX or US actuated NPep_RNP_ particles. Panel a scale bar = 50 µm. Statistical significance determined via one-way ANOVA from n = 3 biologic replicates, with p values shown above relevant comparisons in panel b.

Figure S8. Relative tdTomato signal (in relative fluorescence units, RFU) as a function of vertical height from the base of the organoid. Arrows indicate the peak tdTomato signal for the indicated treatment condition.

Figure S9. Orthogonal views of kidney organoids treated with CRISPRMAX (left) or US actuated NPep_RNP_ (3 W/cm^2^, 50% DC, right). Scale bars = 100 µm.

Figure S10. Percentage of positively stained HEK293 cells following a 4 hour incubation with blank media (untreated), or DiI-loaded NPep particles prepared from peptide surfactants containing either the integrin-targeting RGD sequence or a physiochemically matched RGE binding deficient control. Plots prepared from flow cytometry analysis of n = 3 biologic replicates.

Figure S11. Calibration curve relating NPep particle concentration per μL to solution optical density (OD_600_).
